# Supplementary material for: Comparison of instrument-assisted soft tissue mobilization and proprioceptive neuromuscular stretching on hamstring flexibility in patients with knee osteoarthritis
Source: PeerJ. 2023 Dec 1;11:e16506. doi: 10.7717/peerj.16506 (PMC10695107; doi:10.7717/peerj.16506)
Supplement: Supplemental Information 2 [file peerj-11-16506-s002.docx]

COMPARISON OF ERGON TECHNIQUE VERSUS PNF STRETCHING ON HAMSTRING FLEXIBILITY IN PATIENTS WITH KNEE OSTEOARTHRITIS

SYNOPSIS


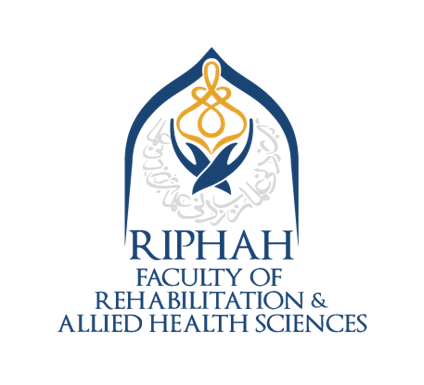


**NARMEEN ANJUM**

**3220**

**Master of Science in Physical Therapy**

**(Orthopedic Manual Physical Therapy)**

**Riphah College Of Rehabilitation &Allied Health Sciences**

**RIPHAH INTERNATIONAL UNIVERSITY ISLAMABAD**

**Fall 2020**

**NARMEENANJUM**

**3220**

**DR. NAZISH RAFIQUE**

**Supervisor**

In Partial Fulfilment of Requirements For the Award of Degree of

Master in Science of Physical Therapy (Orthopaedic Manual Physical Therapy)

**Riphah College of Rehabilitation & Allied Health Sciences**

**RIPHAH INTERNATIONAL UNIVERSITY ISLAMABAD**

**Fall2020**

**RIPHAH INTERNATIONAL UNIVERSITY**

**ACADEMIC PROGRESS REPORT**

**As on**  **MS-PT (OMPT)**

**For the period *from* Fall 2020 to 2022**

1. **Personal Information of Scholar:**

| Name: | Narmeen Anjum |
| --- | --- |
| Registration No.: | 3220 |
| Program: | MSPT(OMPT) |
| Faculty/Department: | Riphah College of Rehabilitation& Allied Health Sciences |
| Email: | [narmeenanjum3@gmail.com](mailto:narmeenanjum3@gmail.com) |

1. **Academic Progress:**

| Admission Date: | September’2020. |
| --- | --- |
| Status of Coursework (Credit hours completed and remaining): | 2^nd^ Semester in progress. |
| Expected Date of Completion of Research Work: | 6 Months after the approval. |
| Expected Date of Completion of Program: | July’2022. |
| Last GPA and CGPA (Please attach result of each semester): | 3.73 GPA. |

1. **Research Topic:**

| Topic of Research: | Comparison of Ergon technique Versus PNF Stretching on Hamstring Flexibility in Patients with Knee Osteoarthritis |
| --- | --- |
| No of article reviewed for synopsis | 17 |
| Date of Approval | 2^nd^ June 2021 |
| Name of Supervisor | Dr. Nazish Rafique. |
| Name of Co-Supervisor (if any): | None |
| Status of Research Work | In progress. |

1. **Employment Status:**

| Unemployed | N/A. |
| --- | --- |
| Employed (job place, title, and status—i.e., on study leave or otherwise) | Al-Ihsan Hospital Rawalpindi. |

*Please Note: The scholars under HEC Indigenous 5000 Fellowship Program shall not undertake any employment whether paid or otherwise at any stage during their course of study of the program.*

Dated: _________________________ Signature of Scholar: ___________________

**Remarks of the Supervisor:**

|  |
| --- |
|  |
|  |

1. **Overall progress: (please tick only one)**

| **Poor** | **Satisfactory** | **Good** | **Very Good** | **Excellent** |
| --- | --- | --- | --- | --- |
|  |  |  |  |  |

| **Verified/Certified by** | **Countersigned by** |
| --- | --- |
| **Supervisor** | **Associate Dean PG&R** |
| Name: Dr. Nazish Rafique | Name: Prof. Waqar Ahmed Awan |
| Signature: | Signature: |
| Date: 6^th^ June 2021 | Date: |

**SIGNATURES**

**Comparison of Ergon technique Versus PNF Stretching on Hamstring Flexibility in Patients with Knee Osteoarthritis**

**Name of Student: Narmeen Anjum**

**Registration No: 3220**

| 1 | Dr. Nazish Rafique | Supervisor |  |
| --- | --- | --- | --- |
| 2 | Dr. | Co-Supervisor |  |
| 3 | Dr. Momena Shehzad | Internal Examiner |  |
| 4 | Dr Sajjad Ali | RTC Member |  |

# INTODUCTION & LITERATURE REVIEW

The proportion of people affected with symptomatic knee OA is likely to increase due to the aging of the population and the rate of obesity or overweight in the general population. About 13% of women and 10% of men aged 60 years and older have symptomatic knee OA. About 10% of people aged over 55 years have painful disabling knee OA of whom one quarter is severely disabled. Prevalence of knee OA in men is lower compared with women. (1) 86.7% of people in the world (20 years and older) developed knee OA in 2020. (2) According to the studies, knee osteoarthritis (KOA) affects 13.6 percent of people in China, 5.78 percent population in India and 10.20 percent people in Bangladesh. According to a study conducted in Pakistan, knee osteoarthritis affects 28.0% of the urban population and 25.0% of the rural population (KOA). (3)

According to World Health Organization, Osteoarthritis (OA) is regarded a major public health problem. It is the major causes for impaired function that decreases quality of life (QOL) worldwide (4) and it is a degenerative joint disease that causes functional impairment and reduced quality of life. (5) Age, sex, genetic predisposition, prior joint trauma, and abnormal mechanical forces, mainly caused by obesity, all play a role in the development of osteoarthritis. (3) The pathogenesis of OA involves a degradation of cartilage and remodeling of bone due to an active response of chondrocytes in the articular cartilage and the inflammatory cells in the surrounding tissues. The release of enzymes from these cells breaks down collagen and proteoglycans, destroying the articular cartilage. The exposure of the underlying subchondral bone results in sclerosis, followed by reactive remodeling changes that lead to the formation of osteophytes and subchondral bone cysts. The joint space is progressively lost over time.

The primary symptoms of OA seen are joint pain, stiffness and limitation of movement. The capacity of a muscle to lengthen and enable a joint to move through a range of motion (ROM) is known as flexibility. (6) In the first and second grades of knee osteoarthritis, muscular tightness (or) protective muscle spasm occurs in the knee musculature, with the extensors of the knee joint quadriceps weakening and reduced hamstring flexibility. (4)

The specific programs that are typically run are a combination of programs such as strength training, aquatic, Tai Chi, aerobic, and hydrotherapy. Strength training, being the most common treatment approach for the management of patients with functional limitations, is prescribed to address the need to increase muscular strength and joint stability for improving WOMAC pain scores and quality of life. There is role of hydrotherapy in aiding normal walking and relieving joint pain. (6) Several adjunct therapies are used to treat knee OA treatments with the goal of maximizing outcomes for patients. Thermal modalities, laser therapy, therapeutic ultrasound, electrical stimulation, manual therapy techniques, taping, acupuncture, among others, are some interventions that are commonly used. (7)

Soft tissue mobilization with the Graston instrument has recently been shown to be successful in promoting hamstring extensibility in shortened hamstrings. (8) By releasing adhesions with stainless steel tools and eliciting a local inflammatory response, this technique is designed to facilitate connective tissue remodeling. Techniques such as proprioceptive neuromuscular facilitation (PNF), static stretching, and active release technique have also been shown to lengthen hamstrings. The physiological reflex mechanism that activates the Golgi tendon organ and causes autogenic inhibition in the agonist muscle is the basis of PNF stretching. (9)

Tahir Mahmood et al. in 2021 showed in his research work that IASTM is successful for early recovery. By using such instrumental techniques, the risk of discomfort and pain associated with overuse of manual techniques can be avoided. This technique can help to prevent musculoskeletal disorders by requiring less force when using mechanical force through instruments.(10)

Meena.V et al. in 2016 concluded in a research that PNF Hold relax stretching combined with moist heat improved pain relief and hamstring flexibility more than static stretching combined with moist heat.(4)

Joseph Paul concluded in his study that M2T blade IASTM combined with traditional physiotherapy improved VAS, WOMAC, and knee ROM better than conventional physiotherapy alone.(11)

Harrison K et al. in 2020 contrasted the efficacy of IASTM and Massage with PNF Stretching (MAS/PNF) in enhancing hamstring muscle tightness and subjective reporting of tightness in physically active individuals. The IASTM group recorded significantly higher levels of discomfort during treatment delivery; however, the discomfort was mild and not severe at 24 and 48 hours after treatment.(12)

Leanna concluded that IASTM is effective for treating tight hamstring muscles and PNF is effective not only for improving passive ROM but also for improving active ROM. This study was done in university students with small sample size. Age group for recruitment of participants was also different. Hip flexion range of motion was measured with a passive straight leg raise (for IASTM) or active straight leg raise (for PNF) before and after stretching. Participants performed a self-static stretch on one leg and received the alternative intervention on the contralateral leg. The two studies were analyzed separately for reliability indices and significant differences between interventions. (9)

Previous studies were done on young population with age group between 18-24 years no history of hamstring pathology, no long term follow up and small sample size. This study will focus on age group between 35-50 years in which people are more susceptible to develop osteoarthritis. This study will find the effects of Graston technique on hamstring flexibility in knee OA, as there was very little research work done in Pakistan on Graston technique.

# 2. OBJECTIVE &HYPOTHESIS OF STUDY

## Objectives

To compare the effects of Ergon technique and proprioceptive neuromuscular facilitation (PNF) stretching for improving hamstring flexibility in patient with knee osteoarthritis

## Hypothesis

- - 1. **True Hypothesis:** There will have effects of Ergon technique and PNF stretching for improving hamstring flexibility in patients with Knee Osteoarthritis.
    2. **Null Hypothesis:** There will be no effects of Ergon technique and PNF stretching for improving hamstring flexibility in patients with Knee Osteoarthritis.

# 3. MATERIAL &METHODS

## 3.1. Study Design

It will be a Randomized Control Trial with two treatment groups. Group A: (Ergon technique) and Group B: (PNF stretching). Randomization will be done by sealed envelope method.

**3.2. Sample Size**


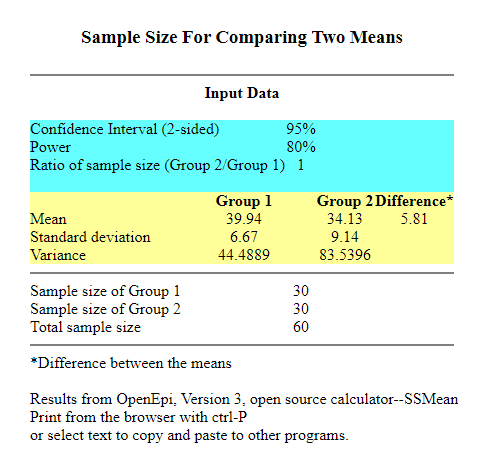
This study would include total 60 participants which would be divided into two treatment groups. 30participants will be included in each group. Sample size was calculated by open Epitool.(12)

.

**3.3. Study Duration**

The study will be conducted for 6 months duration after the approval of the research board.

**3.4. Sampling Technique**

Non probability Purposive Sampling will be used

**3.5. Study Setting**

The study will be conducted at Railway General Hospital Rawalpindi.

**3.6. Selection Criteria**

## . Inclusion Criteria

Participants falling in the category below will be included into the study:

- Age: 35 - 50years
- Both genders
- Unilateral/bilateral OA
- Participants with unilateral/bilateral hamstring tightness
- >20^0^ from AKE test
- Grade 1 and 2 in Kellegren and Lawrence criteria for knee OA

## 3.62. Exclusion Criteria

Participant failing to fall in the category below will be excluded from the study:

- Any lower extremity injury in past 3 months
- Any fracture or surgery done for pelvis, hip or knee.
- Any neurological symptoms.
- Any recent knee reconstructive surgery
- Burns of lower extremities
- Other musculoskeletal disorders associated with knee joint, IT band, adductor muscle and Sartorius muscle tightness

## 3.7. Tools

**3.71. Visual Analog Scale (VAS)**

The visual analogue scale or visual analog scale (VAS) is a measurement instrument for subjective characteristics or attitudes that cannot be directly measured. When responding to a VAS item, respondents specify their level of agreement to a statement by indicating a position along a continuous line between two end-points. The visual analog scale (VAS) is a validated, subjective measure for acute and chronic pain. Scores are recorded by making a handwritten mark on a 10-cm line that represents a continuum between “no pain” and “worst pain. It is used to find out the intensity of pain.(13)

## 3.72. Contoured stainless steel instrument

Graston Technique, instrument assisted soft tissue mobilization (IASTM) will be applied to the posterior thigh with a contoured stainless steel instrument.

## 3.73. WOMAC

The Western Ontario and McMaster Universities Arthritis Index (WOMAC) is widely used in the evaluation of Hip and Knee Osteoarthritis. It is a self-administered questionnaire consisting of 24 items divided into 3 subscales. (14)

**3.74. AKE test**

The Active Knee Extension Test is used to assess hamstring muscle length and the range of active knee extension in the position of hip flexion**.**(15)

The participant is asked to lie supine with 90◦hip and knee flexion and neutral ankle joint position so that the thigh is vertical and the lower leg is horizontal. A pelvic strap was applied around the anterior superior iliac spine (ASIS) to prevent any compensatory movement. The participant then extends the knee and the knee angle is measured using a goniometer (16)

**
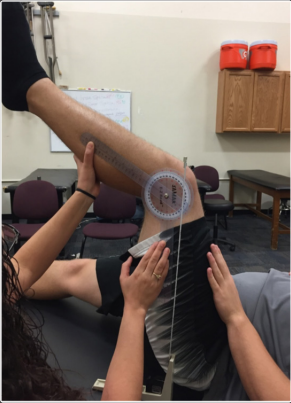
**

## 3.8. Intervention

Participants will be randomly divided in two treatment groups i.e. Group A and Group B through sealed envelope method. Hot pack for 15 min will be applied for 15 minutes before giving the intervention. Ergon technique will be applied to group A along with strengthening exercise. Proprioceptive Neuromuscular Facilitation (PNF) stretching will be given to group B along with strengthening exercise. A 35 minute session will be given to the patients and 3 sessions will be given once a week. Intervention will be given for 6 weeks. (Table 1)

Table 1: Detail Intervention Protocol

| **Treatment duration and frequency** | - 35 minute session will be given to the patients - 3 sessions/week for 6 weeks |
| --- | --- |
| **Group A (ERGON technique)** | - Hot pack will be applied for 15 minutes. - **Patient position:** Patient will be asked to lie prone with knee flexion approximately 45◦. - **Position of therapist:** Therapist will be standing at the affected side of patient. - **Technique:** Massage cream will be applied on the hamstring and overactive or short muscle fibres or bands that are restricted will be identified. The instrument will be applied parallel to the muscle fibers, both in a distal to proximal and them proximal to distal direction as needed to address restrictions Then gentle massage stroking from the origin to the insertion will be performed using the instrument without causing any discomfort or pain. IASTM will be applied for 5 minutes using contoured stainless steel instrument. (16) |
| **Group B (PNF stretching)** | - The patient will be laid down prone and moist heat will be given for 15 minutes prior to stretching the hamstrings. - Then patient will be lying supine with hip in 90⁰ of flexion. Patient’s knee will be extended until a very mild stretching sensation is felt in hamstring muscles. - Then patient will be asked to flex the knee against the resistance applied by hand of therapist. - The patient will be asked to use a force of around 50% of maximal strength and an isometric form of contraction will be gained in the hamstring muscles. The patient will hold the contraction for 8 seconds and then the therapist command to relax the hamstring muscle. - Immediately after the muscle relaxation the therapist further stretches the hamstring muscles up to a point where the subject reported a mild to moderate stretching sensation without any pain and will be held for 30 seconds. - The procedure will be repeated **3 times** in every session. (4) |
| **Strengthening exercises** | - Strengthening exercises for quadriceps and hamstring will then be performed in both groups - **Isometrics exercises of Quadriceps:** By placing a towel roll below the knee and press and hold for 20 seconds, 5 sets. - **Isometrics exercises Hamstrings:** By placing a towel roll below the knee and press and hold for 20 seconds, 5 sets. - **Wall sits:** For 10 seconds, 5 sets. (17) |

## 3.9. **Data Collection Procedure**


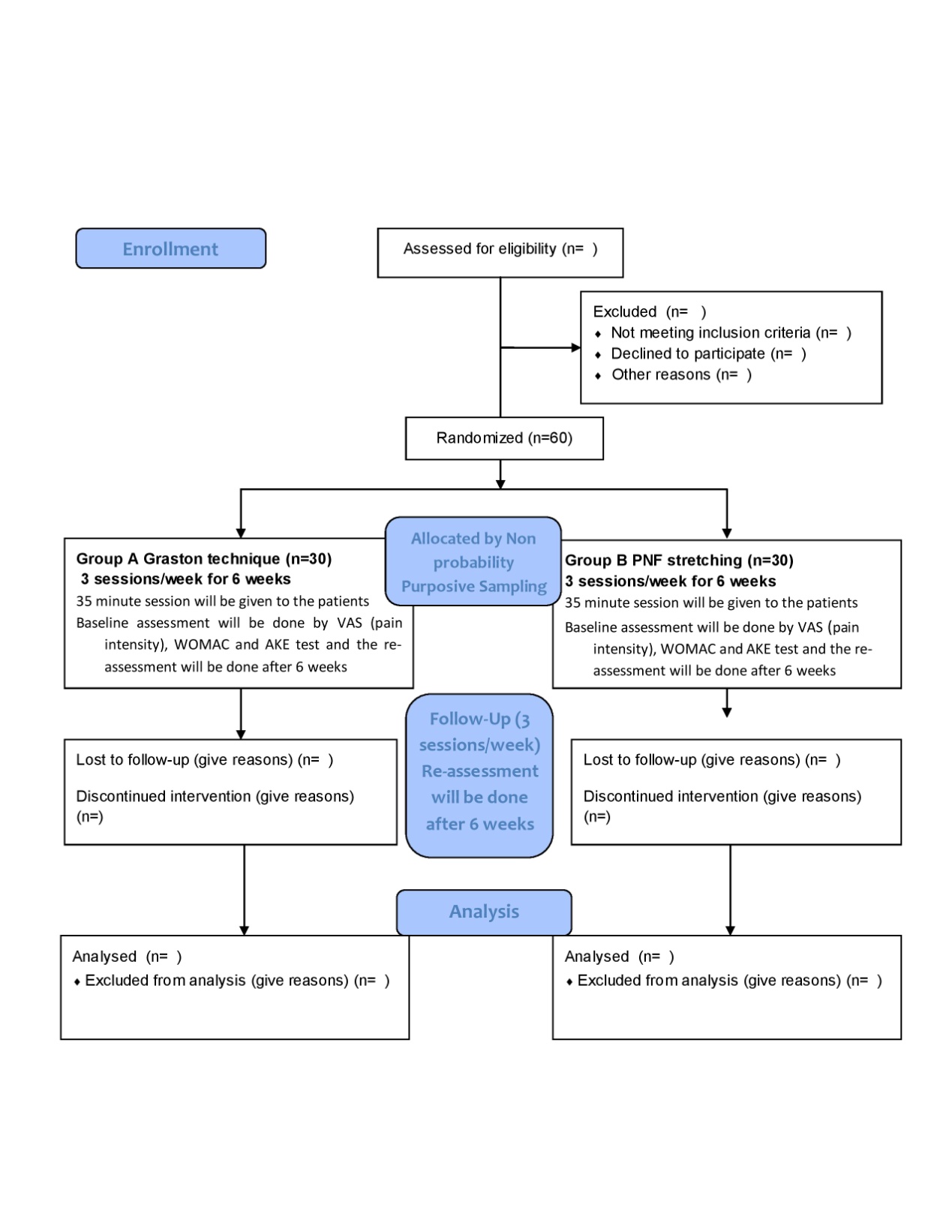


Figure 1: Consort Flow Diagram

## 3.10. Data analysis

Data will be analyzed using SPSS version 23.

## 3.11 Gant Chart

|  | **April-21** | **June-21** | **July-21** | **Aug-21** | **Sep-21** | **Oct-21** | **Nov-22** | **Dec-21** | **Jan-22** |
| --- | --- | --- | --- | --- | --- | --- | --- | --- | --- |
| **Title approval** |  |  |  |  |  |  |  |  |  |
| **Synopsis Defence** |  |  |  |  |  |  |  |  |  |
| **BASR Approval** |  |  |  |  |  |  |  |  |  |
| **Data collection** |  |  |  |  |  |  |  |  |  |
| **Report writing** |  |  |  |  |  |  |  |  |  |
| **Final presentation** |  |  |  |  |  |  |  |  |  |
| **Report submission** |  |  |  |  |  |  |  |  |  |

# REFERENCES

1. Heidari B. Knee osteoarthritis prevalence, risk factors, pathogenesis and features: Part I. Caspian journal of internal medicine. 2011;2(2):205.

2. Cui A, Li H, Wang D, Zhong J, Chen Y, Lu H. Global, regional prevalence, incidence and risk factors of knee osteoarthritis in population-based studies. EClinicalMedicine. 2020;29:100587.

3. Iqbal MN, Haidri FR, Motiani B, Mannan A. Frequency of factors associated with knee osteoarthritis. JPMA-Journal of the Pakistan Medical Association. 2011;61(8):786.

4. Meena V, Shanthi C, Madhavi K. Effectiveness of PNF stretching versus static stretching on pain and hamstring flexibility following moist heat in individuals with knee osteoarthritis. International Journal of Physiotherapy. 2016;3(5):479-84.

5. Jyoti JS, Yadav V. Knee joint muscle flexibility in knee osteoarthritis patients and healthy individuals. Int J Health Sci Res. 2019;9(6):156-63.

6. Onigbinde AT, Akindoyi O, Faremi FA, Okonji A, Shuaib O, Lanre OO. An assessment of hamstring flexibility of subjects with knee osteoarthritis and their age matched control. Clin Med Res. 2013;2(6):121-5.

7. Bhatia D, Bejarano T, Novo M. Current interventions in the management of knee osteoarthritis. Journal of pharmacy & bioallied sciences. 2013;5(1):30.

8. Moon JH, Jung J-H, Won YS, Cho H-Y. Immediate effects of Graston Technique on hamstring muscle extensibility and pain intensity in patients with nonspecific low back pain. Journal of physical therapy science. 2017;29(2):224-7.

9. Gunn LJ, Stewart JC, Morgan B, Metts ST, Magnuson JM, Iglowski NJ, et al. Instrument-assisted soft tissue mobilization and proprioceptive neuromuscular facilitation techniques improve hamstring flexibility better than static stretching alone: a randomized clinical trial. Journal of Manual & Manipulative Therapy. 2019;27(1):15-23.

10. Mahmood T, Hafeez M, Ghauri MW, Salam A. Instrument assisted soft tissue mobilization-an emerging trend 3 for soft tissue dysfunction 4. Journal of the Pakistan Medical Association. 2020:1-14.

11. Coviello JP, Kakar RS, Reynolds TJ. Short‐term effects of instrument‐assisted soft tissue mobilization on pain free range of motion in a weightlifter with subacromial pain syndrome. International journal of sports physical therapy. 2017;12(1):144.

12. Harrison K CM, Dietrich W, Mc Devitt J, Vanic K and Rozea G. The Use of Instrument Assisted Soft Tissue Mobilization Verse Massage and

Proprioceptive Neuromuscular Facilitation Stretching Techniques on Improving

Hamstring Flexibility. International Journal of Orthopaedics Research. 2020;3(1):11.

13. Delgado DA, Lambert BS, Boutris N, McCulloch PC, Robbins AB, Moreno MR, et al. Validation of digital visual analog scale pain scoring with a traditional paper-based visual analog scale in adults. Journal of the American Academy of Orthopaedic Surgeons Global research & reviews. 2018;2(3).

14. Salaffi F, Leardini G, Canesi B, Mannoni A, Fioravanti A, Caporali Ro, et al. Reliability and validity of the Western Ontario and McMaster Universities (WOMAC) Osteoarthritis Index in Italian patients with osteoarthritis of the knee. Osteoarthritis and cartilage. 2003;11(8):551-60.

15. Hamid MSA, Ali MRM, Yusof A. Interrater and intrarater reliability of the active knee extension (AKE) test among healthy adults. Journal of physical therapy science. 2013;25(8):957-61.

16. Kim D-H, Lee JJ. Effects of instrument-assisted soft tissue mobilization technique on strength, knee joint passive stiffness, and pain threshold in hamstring shortness. Journal of back and musculoskeletal rehabilitation. 2018;31(6):1169-76.

17. Ganesh B. Mpt PhD D, Pathan Bpt A, Tiwade Bpt A. Short term effects of instrument assisted soft tissue mobilization on pain and activities of daily living in subjects with patellofemoral joint osteoarthritis – A randomized controlled trial. International Journal of Current Research in Medical Sciences. 2017;4(11):55-63.

**ANNEXURES**

**Anexure 1: Consent form (English)**

**
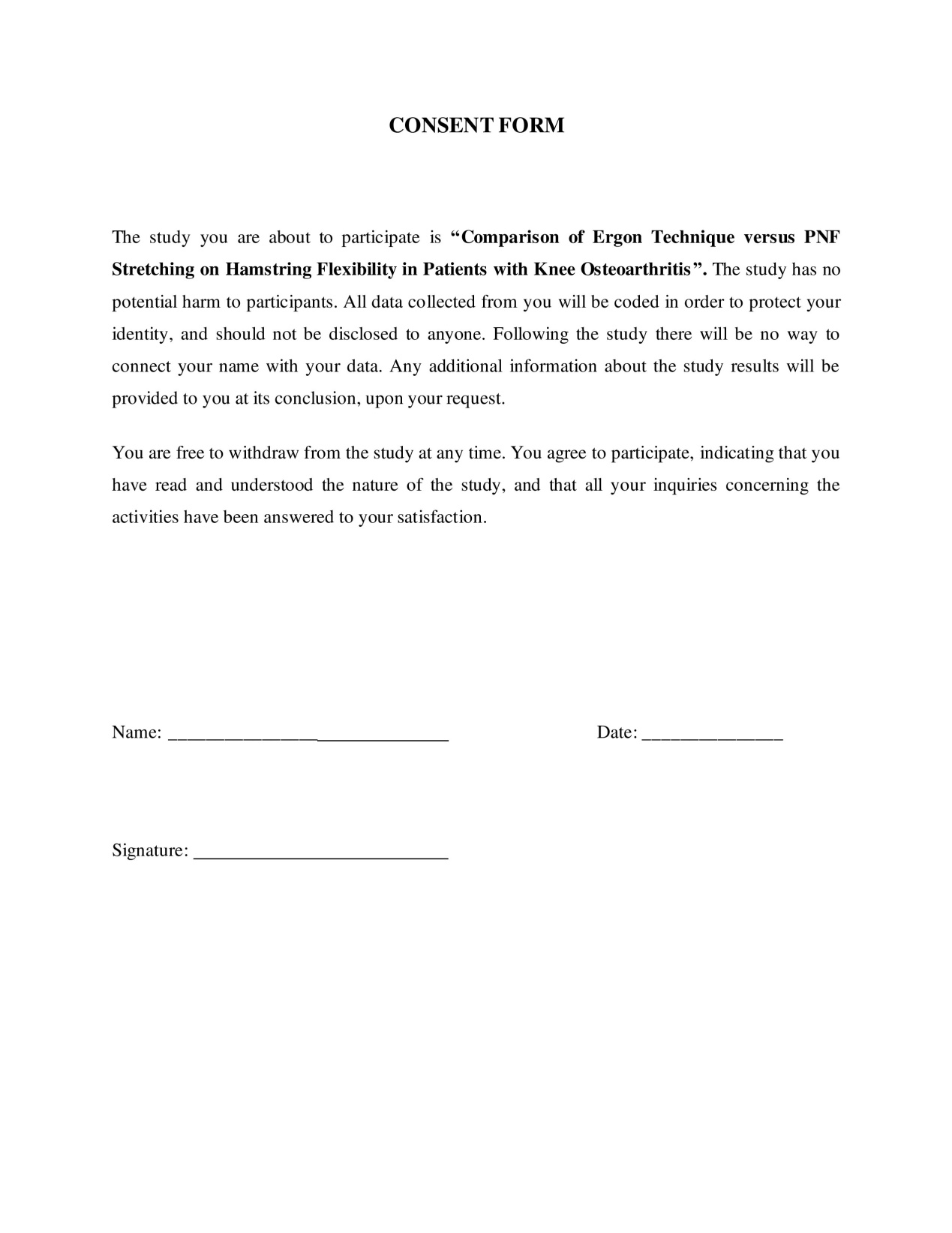
Anexure 2: Consent form (Urdu)**


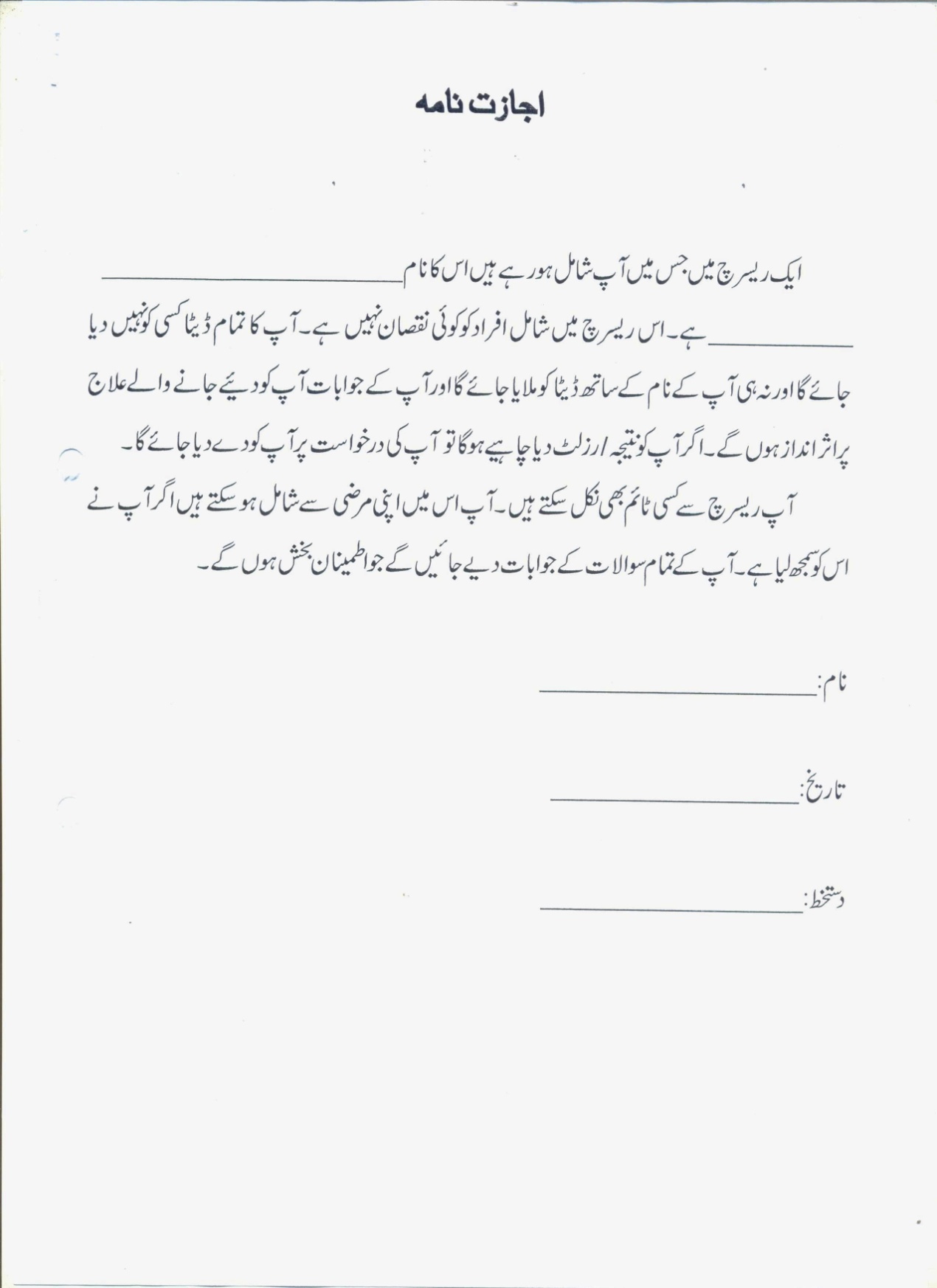
**Anexure 3: Demographics form & Questionnaires**

**Comparison of Ergon Technique versus PNF Stretching on Hamstring Flexibility in Patients with Knee Osteoarthritis**

**Section 1**

**Demographics History:**

**Name: ______________________ Age:**_______ **Gender:** a) Male b) Female

**Marital status:** a) Married b) Unmarried c) Widow d) Divorced

**Education:** a) No formal education b) Up to primary c) Up to secondary d) Above secondary

**Weight (kg): _________ Height (cm): ___________ BMI: (kg/m2):________**

**History of knee pain: ______________**

**Section 2**

**Osteoarthritis related questions:**

**1. Do you have unilateral or bilateral knee OA?**

a) Unilateral b) Bilateral

**2. Grade of Knee Osteoarthritis according to Kellegren and Lawrence criteria:**

a) Grade 1 b) Grade 2

**SECTION 3**

**Tools:**

| **Variables** | | **Baseline Assessment** | **Assessment at 6^th^ Week** |
| --- | --- | --- | --- |
| **AKE Test** | **Right** |  |  |
|  | **Left** |  |  |
| **VAS** | |  |  |
| **WOMAC** | |  |  |

**Visual Analog Scale**


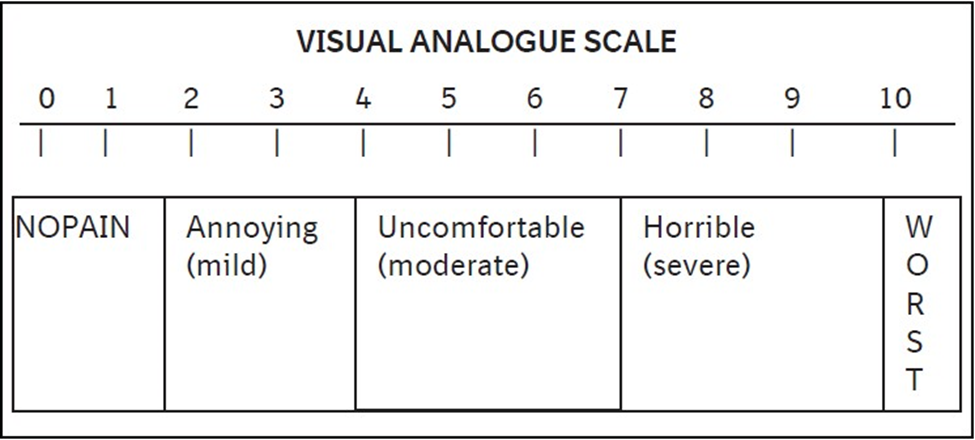


**
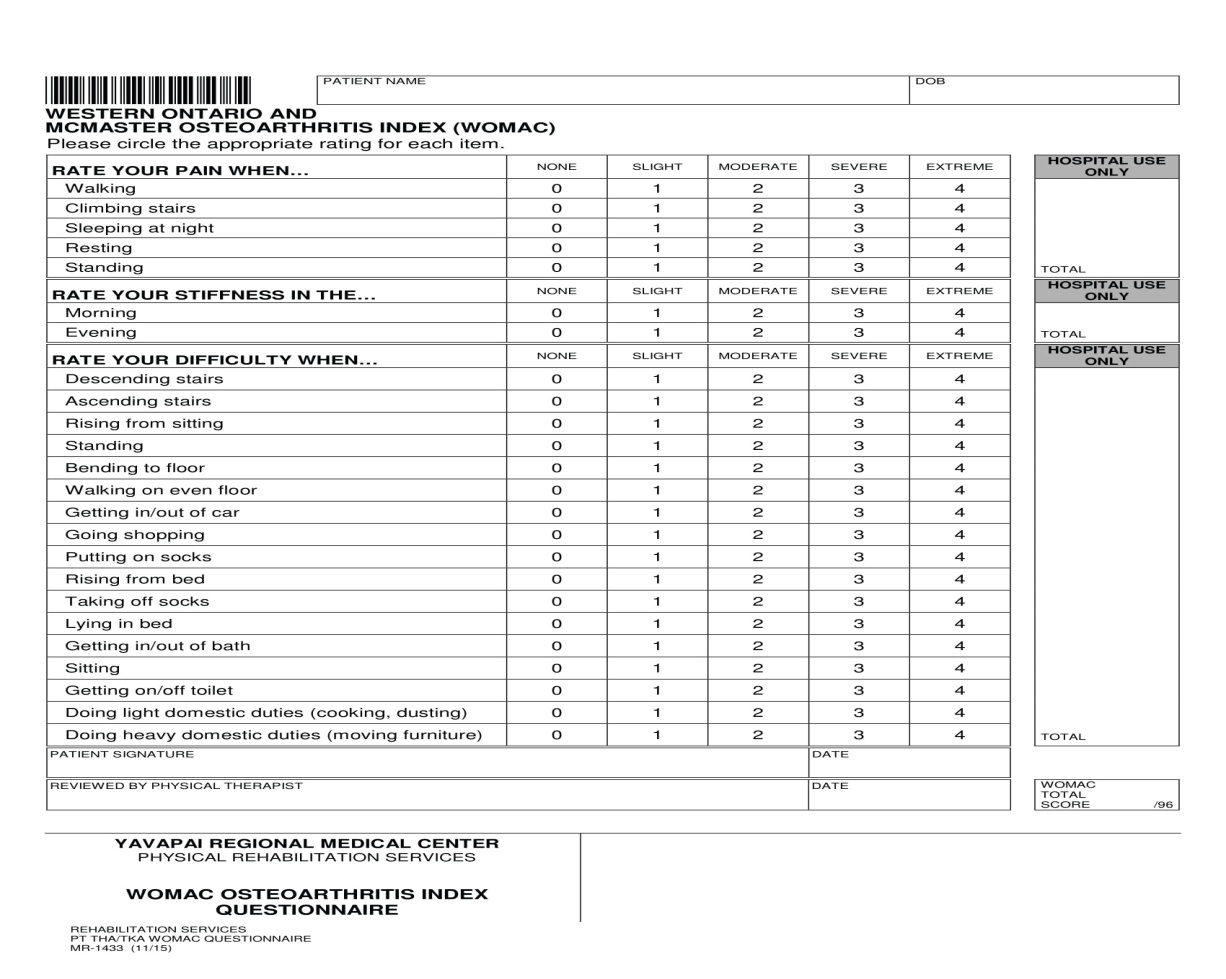
 Western Ontario and McMaster Universities Osteoarthritis Index (WOMAC)**

**Anexure : Permission Letter**

**
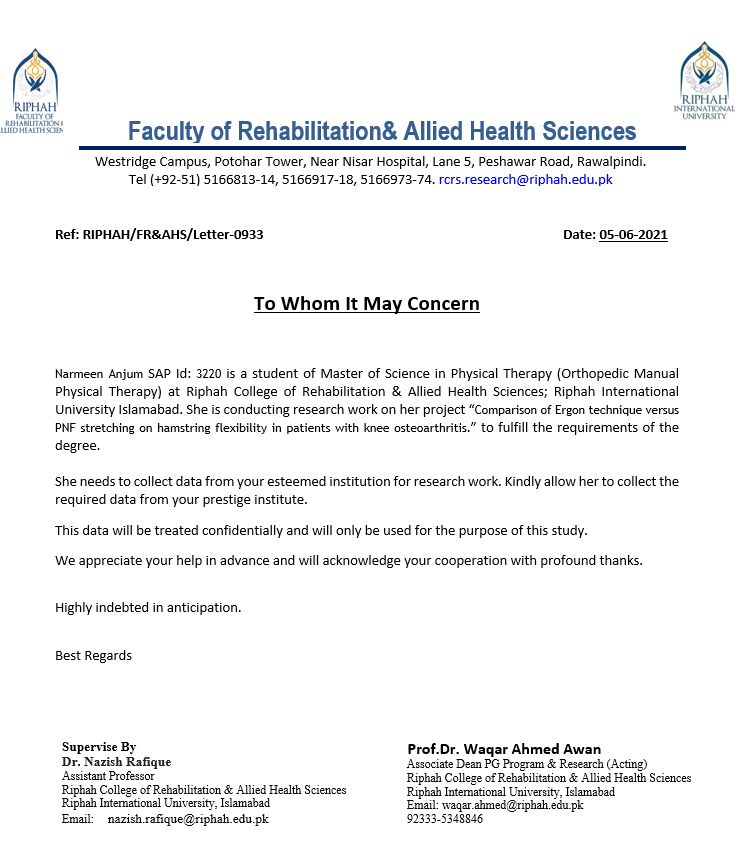
**
